# Deaths

**Published:** 1781-02

**Authors:** 


### DEATHS.

*Nov.* 29, 1780, at Leyden, aged 76 years, Jerom David Gaubius, M. D. Honorary Profeſſor of Chemiſtry and the Practice of Phyſic, Firſt Phyſician to the Stadtholder, F. R. S. of London, and of many other ſimilar inſtitutions in different parts of Europe. He was a native of Heidelberg in Germany, and was the favourite pupil of Boerhaave, who reſigned the chemical chair in his favour in 1735, as he himſelf did in 1775 in favour of his nephew Dr. Hahn. His works are few in number, but his *Inſtitutiones*

*Pathologiæ Medicinales* alone are a sufficient proof of his great abilities as a medical writer.

*Dec.*—At Vienna, aged 27 years, Maximilian Fellner, M. D. Professor of Physiology in the University at that place.

6.—At Versailles, aged 78, Joseph Lieutaud, M. D. Counsellor of State, and First Physician to the King of France, Member of the Royal Academy of Sciences at Paris, of the Royal Society of London, &c. He was a native of Aix in Provence, and in 1774 succeeded M. Senac as archiater. He acquired considerable reputation as a practical writer by his *Synopsis Universæ Praxeos Medicæ*, 2 vols. 8vo.; and as an anatomist, by his *Essais Anatomiques* (a late edition of which is much improved by M. Portal) and his *Historia Anatomico-Medica sistens numerosissima cadaverum humanorum extispicia*. 2 vols. 4to.

*Feb.*—1781.—At Chesterfield in Derbyshire, Mr. John Willot, surgeon and apothecary.

15.—At Darlington, in the county of Durham, John Trotter, M. D. author of an inaugural discourse, "*de Morbo Venereo*," printed at Leyden in 1757.

